# Temperature Dependence and Microstructure Effects on Magnetic Properties of FePt(B, Ag, C) Film

**DOI:** 10.3390/nano11020419

**Published:** 2021-02-07

**Authors:** Jai-Lin Tsai, Shi-Min Weng, Cheng Dai, Jyun-You Chen, Xue-Chang Lu, Ting-Wei Hsu

**Affiliations:** Department of Materials Science and Engineering, National Chung Hsing University, Taichung 402, Taiwan; g107066065@mail.nchu.edu.tw (S.-M.W.); g106066063@mail.nchu.edu.tw (C.D.); g106066003@mail.nchu.edu.tw (J.-Y.C.); g108066057@mail.nchu.edu.tw (X.-C.L.); g106066043@mail.nchu.edu.tw (T.-W.H.)

**Keywords:** perpendicular magnetic anisotropy, coercivity, switching field distribution, intergranular exchange coupling, magnetic cluster size, grain size

## Abstract

A FePt(B, Ag, C) granular film was formed from post-annealed B_4_C(1.0 nm)/FePt(Ag, C) layers at a substrate temperature of 470 °C for 2 min. The 6 nm thick FePt(B, Ag, C) film demonstrates high perpendicular magnetic anisotropy (K_u_ = 2.83 × 10^7^ erg/cm^3^ at 100 K) and out-of-plane coercivity (H_c_ = 38.0 kOe at 100 K). The K_u_ and out-of-plane H_c_ are respectively increased from 38% and 46% between 350 K and 50 K. The sample with a thickness of 8 nm also shows a similar trend for magnetic properties; however, the tiny magnetization kink which may come from rare Fe-B or disordered FePt grains was observed in the easy axis loop. The intrinsic (ΔH_int_ = 12.6 kOe) and extrinsic switching field distribution (ΔH_ext_ = 1.62 kOe) were characterized by major and minor loops to correlate the microstructural grains. The coupled FePt grains grown on a single MgTiON grain were observed in a high-resolution transmission electron microstructure (HRTEM) image. This small intergranular exchange coupling was defined by estimating the magnetic cluster size (46.6 nm) from ΔH_ext_ and the average grains size (28.2 nm) from TEM images. The temperature dependence of coercivity was fitted to further understand the magnetization reversal process. The lower microstructural parameter was evidenced in the imperfect grain morphology.

## 1. Introduction

A promising heat-assisted magnetic recording (HAMR) hard drive with L1_0_ FePt media will come to the market soon. The [001] textured FePt film shows a moderate Curie temperature (<750 K) and a high perpendicular magnetocrystalline anisotropy (K_u_) of 5 × 10^7^ erg/cm^3^, which make sure the grains are thermally stable down to 3 nm [1,2,3,4,5]. The advantage of FePt media is its higher area density (~2 Tb/in^2^) than conventional perpendicular magnetic recording (PMR). To ensure much higher area density in the future, the FePt media needs to have a specific microstructure, and tunable intrinsic and extrinsic magnetic properties. Intrinsically, the ordered L1_0_ FePt grains with out-of-plane c-axis alignment were required to promote high-perpendicular magnetic anisotropy with a lower in-plane magnetic component. High-temperature deposition and annealing are an appropriate method to achieve high-ordered FePt film, but they also enhance the growth of the grains. To avoid coarse grains, suitable segregants are necessary to separate and refine the FePt grains in a uniformly granular and columnar structure.

The B_4_C compound was used as an interlayer, a capping-layer, and the doping material, together with (Ag, C) segregants [6,7,8], and the [001] textured FePt granular structure was formed on varied MgO-like intermediate layers after high-temperature deposition [9,10,11,12,13,14,15]. In this work, a FePt(B, Ag, C) granular film was formed after the annealing of the capped B_4_C/FePt(Ag, C) dual-layer. The C has a strong phase separation ability for a FePt film and the Ag was used to moderate the ordering degree. After high-temperature deposition and annealing, a 1 nm thick B_4_C layer was diffused out in the atomic B and C in the FePt grains and boundaries, evidenced in electron energy loss spectroscopy (EELS) mapping [16]. The Fe–B compound was observed in the X-ray photoelectron spectroscopy (XRS) spectra and evidenced in the tiny kink of the FePt (8 nm) magnetic hysteresis loops [16].

The FePt(B, Ag, C) granular film was prepared in a novel way by the diffusion of B, N and C atoms between the B_4_C capping layer and the MgTiON underlayer, and the switching field distribution (SFD) behavior of this film has rarely been discussed before. The correlation of grains size and magnetic clusters is investigated and well discussed in this work. Reducing the switching field distribution (SFD), which includes the intrinsic and extrinsic properties of the FePt grains, can improve the media noise [4,17,18]. In this study, the average switching field defined by out-of-plane H_c_ was obtained in the major magnetic hysteresis loop, and the switching field distribution (SFD) was examined to further understand the magnetic characteristic via magnetic minor loops measurement [4,17,18]. The intrinsic part of SFD includes the contribution of anisotropy filed dispersion, out-of-plane c-axis misalignment, and grain size distribution [18]. The grains’ dipole and exchange coupling belong to the extrinsic contribution of SFD [18]. Furthermore, the magnetization reversal process was tested by the linear tendency of the temperature dependence of coercivity, and the microstructural parameter was obtained after linear fitting [19,20,21]. The discussion of a microstructural parameter can be correlated to the FePt grains images.

## 2. Materials and Methods

Magnetron sputtering was used to deposit the B_4_C/FePt(Ag, C)/MgTiON/CrRu films on a glass substrate with the argon working pressure of 10^−2^–10^−3^ Torr. The high vacuum sputtering system was designed with a main chamber background pressure of 1 × 10^−7^ Torr and the load-lock system was used to transfer the substrate via a pre-chamber. The multiple sputtering sources (A320, AJA INTERNATIONAL INC, MA, USA, MAK 2”, US Technologies West, San Jose, CA, USA) with 2 inch diameters were set up on the main chamber and the substrate was heated via a halogen lamp (1000W, OSRAM GmbH, Munich, Germany) with a maximum heating temperature of 800 °C. The glass substrate (Eagle 2000, Corning Display Technologies, Taipei, Taiwan) with the dimension of 1 cm^2^ was cleaned with de-ionized water, acetone, and ethyl alcohol solvents step by step, and each round lasted 30 min with ultrasonic vibration. After cleaning, the substrate was dried under a nitrogen atmosphere.

The target compositions were Fe_52_Pt_48_, Ag, C, (Mg_0.5_Ti_0.5_) (O_0.9_N_0.1_), Cr_83_Ru_17_ and B_4_C, and the diameter was 2 inches. The deposition rate (*v*) and Ar working pressure (P_Ar_) of each layer were set to 0.165 nm/s (60W-DC) (MDX500, Advanced Energy, Fort Collins, CO, USA), P_Ar_ = 3 mTorr for CrRu; 0.045 nm/s (100W-RF) (PFG 300 RF, Huettinger Elektronik, Freiburg, Germany), P_Ar_ = 10 mTorr for MgTiON; 0.088 nm/s (30W-DC), P_Ar_ = 3 mTorr for FePt and 0.035 nm/s (27W-DC) (MDX500, Advanced Energy, Fort Collins, CO, USA) for (Ag, C) 40 vol%; 0.022 nm/s (100W-RF) (PFG 300 RF, Huettinger Elektronik, Freiburg, Germany), *P*_Ar_ = 10 mTorr for B_4_C.

The 76 nm thick CrRu seed layer with (002) texture was sputtered at 290 °C on a glass substrate to promote the (002) textured MgTiON intermediate layer, which was deposited at 435 °C for 30 nm. The MgTiON film showed a face-centered cubic (fcc) structure with a lattice constant of 0.422 nm. The lattice parameter of the a-axis was 0.384 nm and the c-axis was 0.372 nm for the FePt film. The lattice misfits between the MgTiON/CrRu and FePt/MgTiON layers were around 3% and 9%, respectively. It is suggested that the MgTiON(002)/[100] layer was grown on a CrRu(002)/[110] seed layer epitaxially [12]. Finally, the L1_0_ FePt-40vol%(Ag, C) layer with thicknesses of 6 and 8 nm were heteroepitaxially grown on MgTiON at 470 °C. The 1 nm thick B_4_C-capped layer was sputtered on the magnetic FePt(Ag, C) layer at 470 °C, and post-annealing was maintained for 2 min. After the diffusion of B, C and N atoms from the respective B_4_C and MgTiON layers, the released B, C and N atoms caused lots of vacancies and defects inside the FePt film, which promoted the ordering degree and increased the coercivity [16]. After high-temperature deposition, the ordered FePt(B, Ag, C) film had a granular structure.

The crystallographic structure, texture, and order parameters of FePt were investigated and estimated by X-ray diffraction (XRD), and the θ/2θ diffraction patterns were collected using a standard X-ray diffractometer (BRUKER, D8 Discover, Bruker, Billerica, MA, USA). Magnetic hysteresis loops in the hard-axis (in-plane) and easy-axis (out-of-plane) directions were measured at room temperature by using a superconducting quantum interference device (SQUID) magnetometer (MPMS-XL, Quantum design, San Diago, CA, USA). The microstructure of the films was quantified by using transmission electron microscopy (TEM, JEOL JEM-2010, Tokyo, Japan).

## 3. Results and Discussion

**Figure 1:**
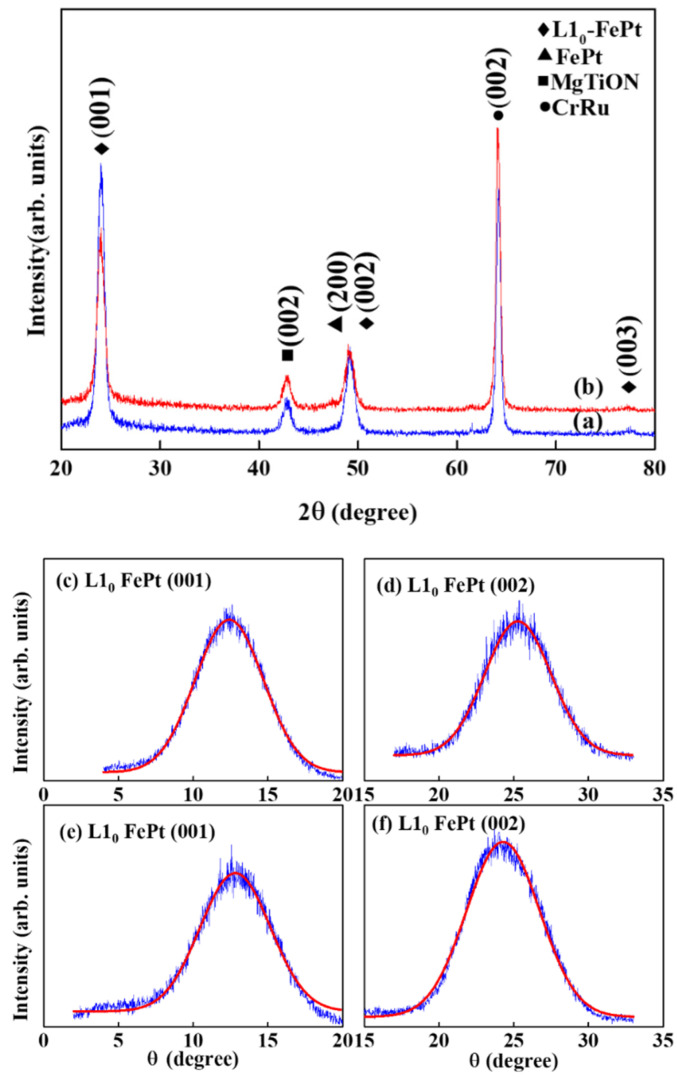
XRD patterns and rocking curves of FePt(B, Ag, C)(*x*)/MoC/MgTiON/CrRu films, *x* = (**a**) 8 nm, (**b**) 6 nm, (**c**,**d**) 8 nm, (**e**,**f**) 6 nm.

Figure 1 shows the XRD pattern of the FePt-(B, Ag, C)(*x* nm)/MgTiON/CrRu films, *x* = (a) 8 (b) 6. The (002) diffraction peaks of the CrRu seed layer and the MgTiON intermediate layer are illustrated, and the (001)/(003) superlattice and (002) fundamental diffraction peaks of the L1_0_ FePt phase are exhibited.

The lattice misfit between FePt/MgTiON is 9%, and the FePt film is under tensile stress during ordering and grain growth. The FePt ordering degree is proportional to the [I(001)/I(002)]^1/2^ ratio; here I(001) and I(002) are the integrated intensities of the FePt (001) and (002) diffraction peaks, and the ratio is 2.99 (the ordering values estimated from [(I_(001)_/I_(002)_)/(I*_(001)_/I*_(002)_)]^1/2^ are 0.89 [22,23] and 2.87, respectively, shown in Figure 1). The rocking curve was measured to evaluate the c-axis alignment of the FePt grains and the width (Δθ_5__0_) was determined by measuring the full width at half maximum (FWHM) of the (001)/(002) reflection peaks; the measured values are 5.9°/5.7° and 5.7°/5.4°, as shown in Figure 1c–f, respectively.

**Figure 2:**
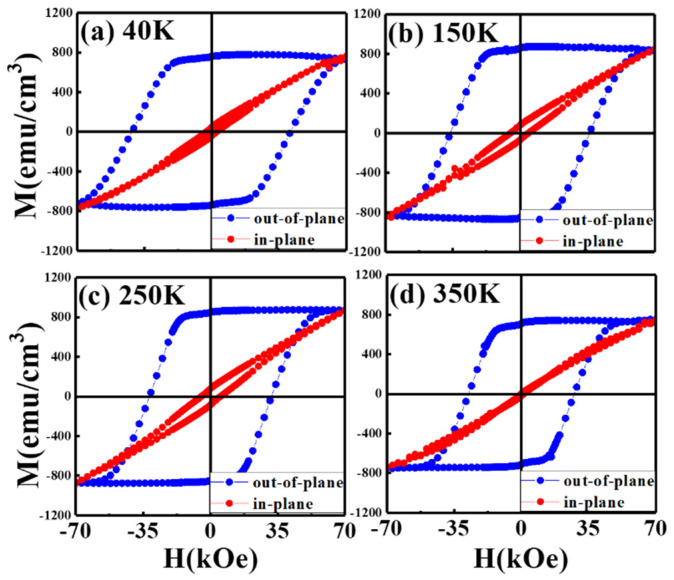
Out-of-plane and in-plane field dependent magnetization loops of the FePt (B, Ag, C)(8 nm)/MoC/MgTiON/CrRu films measured at (**a**) 40 K, (**b**) 150 K, (**c**) 250 K, (**d**) 350 K.

**Figure 3:**
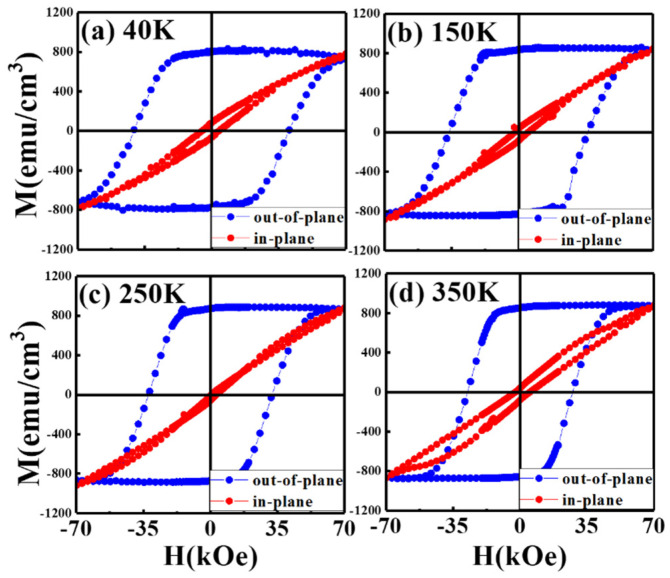
Out-of-plane and in-plane field-dependent magnetization loops of FePt (B, Ag, C) (6 nm)/MoC/MgTiON/CrRu films measured at (**a**) 40 K, (**b**) 150 K, (**c**) 250 K, (**d**) 350 K.

Figure 2 and Figure 3 illustrate the magnetization hysteresis curves in the easy- and hard-magnetic axis of FePt-(B, Ag, C)(*x* nm)/MgTiON/CrRu, *x* = 8 and 6, measured from 40 K to 350 K. Two samples present perpendicular magnetic anisotropy with a high nucleation field in the easy-axis magnetic loops, and the hard-axis loop is linear-like with lower hysteresis.

The saturation magnetization (M_s_) of two samples is around 800 emu/cm^3^, and the remanence ratio (M_r_/M_s_) is almost 1.0 for two samples. When the measured temperature is down to 40 K, the out-of-plane coercivity is around 40 kOe for both samples. Only the 6 nm thick FePt(B, Ag, C) film measured at 350 K shows a wide-up hard-axis loop that may be due to thermal instability.

**Figure 4:**
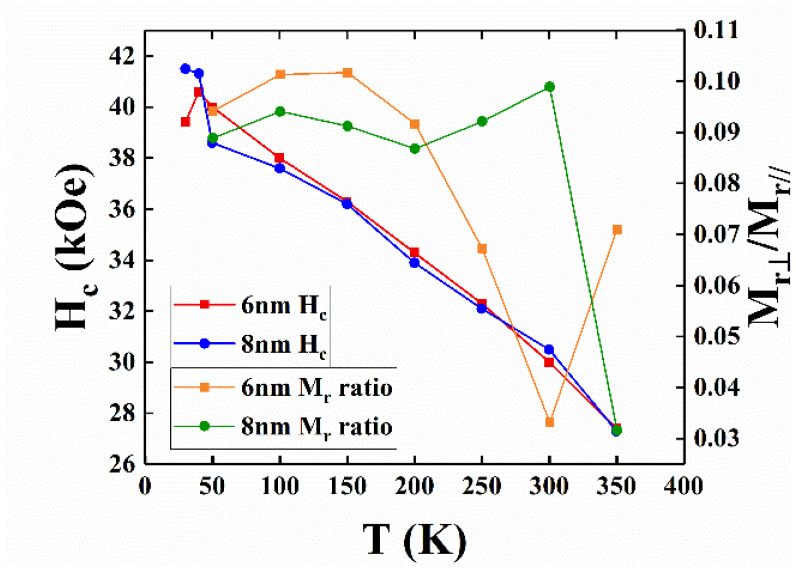
Temperature dependence of out-of-plane coercivity and remanence ratio of FePt (B, Ag, C)/MoC/MgTiON/CrRu films.

The temperature-dependence of out-of-plane coercivity is a plotted in Figure 4. The out-of-plane H_c_ increased with decreasing the measured temperature from 350 K to 40 K, and the differences in the H_c_ values were between 0.1 and 0.5 kOe for the 6nm and 8 nm samples between 100 K and 350 K. The lower H_c_ of the 8 nm sample measured at 50 K was due to the formation of a minor Fe–B compound, and the larger average grain sizes (28.2 nm (for 8 nm sample); >23.3 nm (for 6 nm sample)).

The maximum applied field of 7 T was almost not enough to saturate the sample around 40 K, and the value of coercivity was for reference only. The tiny kink at the zero fields caused by the c-axis misalignment is observed in Figure 2a–d, and is evidenced in the wider rocking width (Δθ_50_ = 5.9°/5.7°) for the FePt-(B, Ag, C)(8 nm)/MgTiON/CrRu sample. The in-plane component was estimated by the ratio of in-plane to out-of-plane remanence (M_r_^(in-plane)^/M_r_^(^°^ut-^°^f-plane)^) [16] and the values measured at different temperatures are plotted in Figure 4. The remanence ratio ranged from 0.03 (3%) to 0.1(10%), and the c-axis of FePt grains aligned in an easy magnetization direction above 90%. The in-plane magnetization and c-axis misalignment may be due to the formation of a rare Fe–B compound or disordered FePt grains.

**Figure 5:**
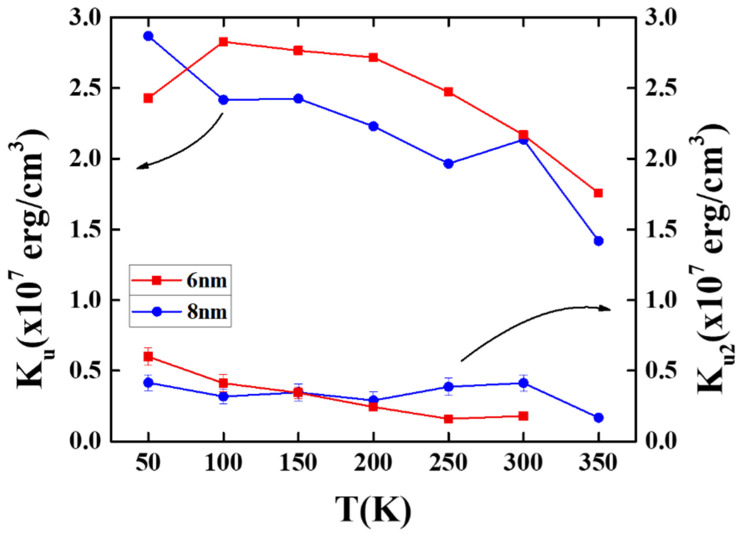
Temperature dependence of the magnetic anisotropy constants (K_u_) and the forth-order anisotropy constant (K_u2_) of FePt (B, Ag, C)/MoC/MgTiON/CrRu films.

Figure 5 shows the temperature dependence of the magnetocrystalline anisotropy constant (K_u_), and the anisotropy constant was determined as K_u_ = H_k_M_s_/2. Here, H_k_ is the anisotropy field estimated by the merge field of the in-plane and out-of-plane magnetization loops.

The L1_0_ FePt-(B, Ag, C) films with thicknesses of 6 nm and 8 nm showed maximum K_u_ values of 2.83 × 10^7^ erg/cm^3^ at 100 K and 2.87 × 10^7^ erg/cm^3^ at 50 K, respectively, and the L1_0_ FePt deposited on MgO(001) showed a higher K_u_ value (>3.5 × 10^7^ erg/cm^3^ in reference [19]), which may due to the lesser amount of interface defects between FePt and the MgO single crystal.

For the 6 nm sample, the lower K_u_ (2.43 × 10^7^ erg/cm^3^) experimentally measured at 50 K was due to a larger error that occurred in the subtraction of the substrate effect of in-plane loops under a limited maximum applied field of 7 T [16]. The K_u_ values decreased with the measured temperature and were down to 1.76 × 10^7^ erg/cm^3^ and 1.41 × 10^7^ erg/cm^3^ at 350 K for the respective 6 nm and 8 nm samples.

**Figure 6:**
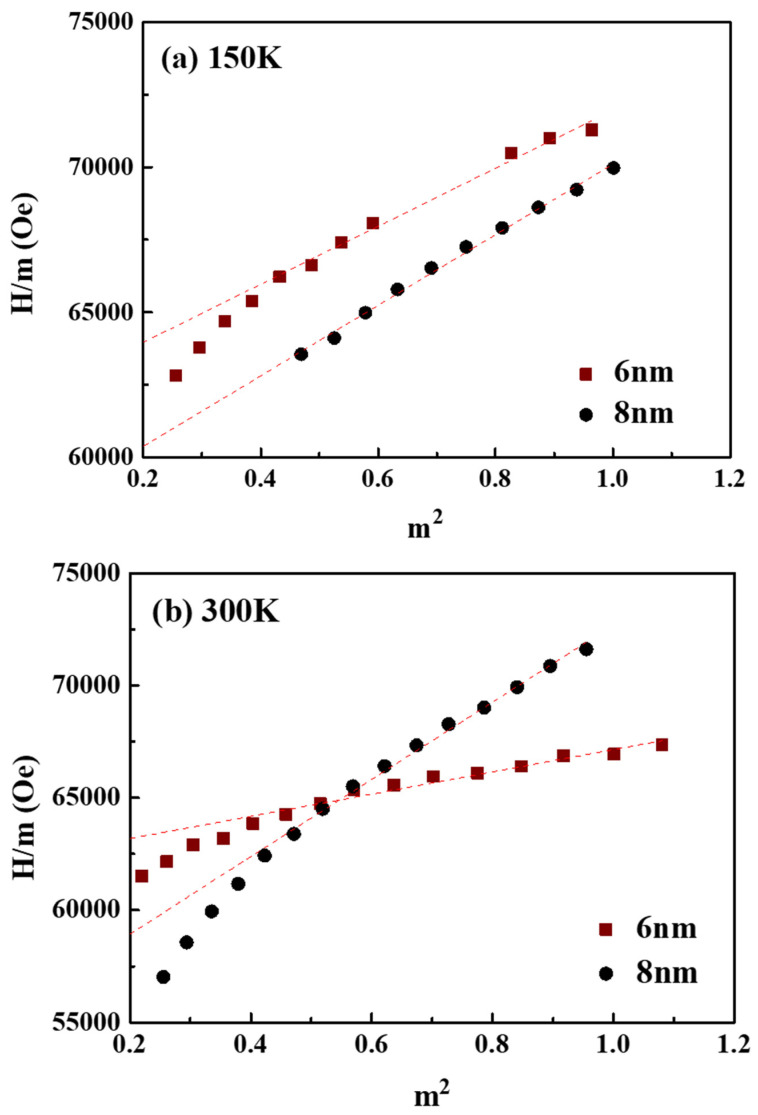
Analysis of the hard-axis loop according to Sucksmith–Thompson for FePt (B, Ag, C)/MoC/MgTiON/CrRu films measured at (**a**) 150 K, (**b**) 300 K.

To disclose the magnetic anisotropy in the 6 nm and 8 nm samples, the second- and forth-order $K_{1}$, and $K_{2}$ anisotropy constants were evaluated by plotting the hard-axis curve individually and shown in Figure 5 and Figure 6 [24,25].

$$\frac{H}{m}=\frac{2K_{1}^{’}}{\mu_{0}M_{s}}+\frac{4K_{2}}{\mu_{0}M_{s}}m^{2}$$

where *m =* $M/M_{s}$ is the reduced magnetization and $K_{1}^{’}$ is the second-order anisotropy constant (Sucksmith–Thompson method). From the *H/m* vs. *m*^2^ plot, the $K_{1}$ and $K_{2}$ constants were determined by the intercept and the slope of the respective fitting curve [24,25]. The *K*_2_/*K*_1_ ratios of the 6 and 8 nm samples were 0.06 (*K*_2_ = 1.79 × 10^6^)/(*K*_1_ = 2.67 × 10^7^) and 0.10 (*K*_2_ = 1.68 × 10^6^)/(*K*_1_ = 1.67 × 10^7^) at 300 K, and they have the same value of 0.14 at 150 K. The small difference in *K*_2_/*K*_1_ ratio in the two samples means a near trend in the angular dependence of the switching field.

**Figure 7:**
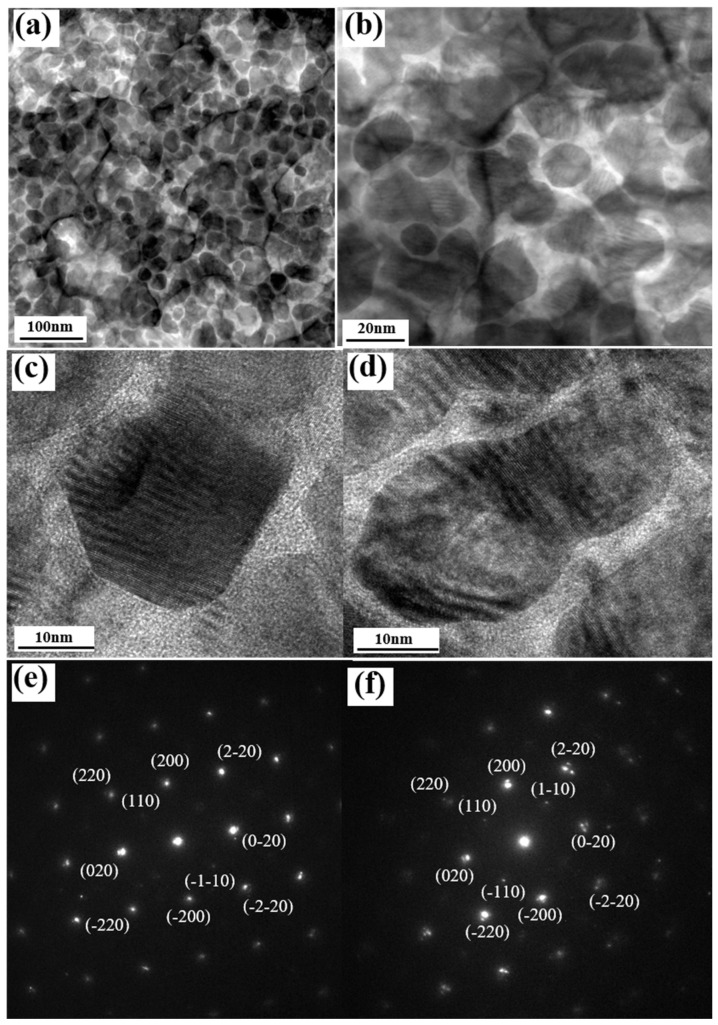
(**a**,**b**) The plane view TEM images of FePt-(B, Ag, C)(8 nm)/MgTiON/CrRu film, (**c**,**d**) magnified TEM images of (**a**,**b**), (**e**) nano-beam diffraction pattern of isolated FePt grain in (**c**), (**f**) nano-beam diffraction pattern of interconnected FePt grain in (**d**).

**Figure 8:**
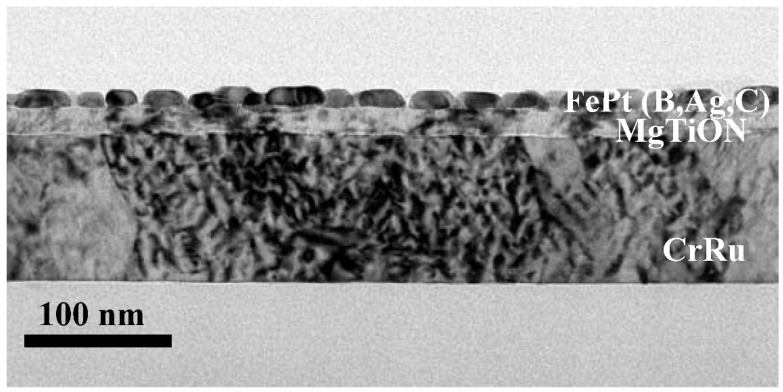
The cross-sectional TEM images of FePt-(B, Ag, C)(8 nm)/MgTiON/CrRu film.

Figure 7 shows the plane view TEM images of the FePt-(B, Ag, C)(8 nm)/MgTiON/CrRu film. In Figure 7a, the FePt grains are separated by the (Ag, C) at the grain boundary areas, and formed the granular structure. The average grain size was 28.2 nm and the average grain boundaries width was estimated by measuring the grains’ pitch δ (i.e., the center-to-center distance) [16], with a value of 3.97 nm. In Figure 7a,b, parts of the FePt grains were grown on the boundaries of MgTiON, and some FePt grains were interconnected in magnetic clusters with lengths in between 47.1 nm and 56.5 nm, which are near to the size of the clusters size (46.4 nm) estimated by Equation (3). The FePt grains’ morphology was polygon and polyhedral, due to the kinetics of grain growth. The lattice fringes in isolated and interconnected FePt grains were observed in Figure 7c,d. The respective average lattice spacings (d_(110)_) were 0.278 nm and 0.283 nm, and the grains’ lattice constant “*a*” was 0.393 nm and 0.400 nm. For the same lattice constant “c” (0.384 nm), shown in Figure 1a, the (c/a) ratios in Figure 7c,d were 0.977 and 0.960, respectively. The interconnected FePt grains in Figure 7d show a higher average ordering degree because the (c/a) ratio is disproportional to the ordering degree. It is suggested that the ordering degree was increased after the FePt grains’ growth and coursing. The grains boundaries were almost amorphous in Figure 7c,d, but some lattice fringe can still be found in the boundary areas of Figure 7d. The nano-beam diffraction patterns of the L1_0_ FePt grains are shown in Figure 7e,f, and the (110), (200) and (220), and related symmetric planes, were indexed in the [001] zone axis. The cross-sectional TEM images of FePt(B, Ag, C)(8 nm)/MgTiON/CrRu are illustrated in Figure 8, and the thickness of each layer was deposited based on the deposition rate and calibrated by the microstructure image.

**Figure 9:**
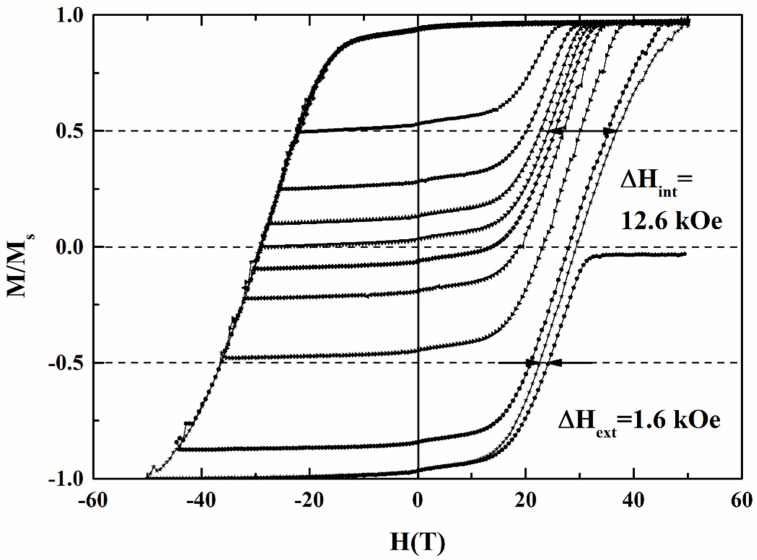
Major and minor loops of the FePt(B, Ag, C)(8 nm)/MgTiON/CrRu film. The minor loop is used to measure intrinsic and extrinsic switching field distribution (SFD).

To understand the correlation between the magnetic switching behavior and grains’ morphology, we analyzed the switching field distribution (SFD), which comes from the intrinsic and extrinsic contribution of magnetic grains. Figure 9 shows the major and minor magnetization loops of the FePt(B, Ag, C)(8 nm) film, and the intrinsic (ΔH_int_ = 12.6 kOe) and extrinsic (ΔH_ext_ = 1.62 kOe) switching field distributions were determined. The recoil loop in the coercivity field means the switching of 50% of the grains in the easy axis, and the demagnetization field between the grains, were neglected. As compared to the major magnetization loop, the parameters ΔH_int_ and ΔH_ext_ were collected. The intrinsic SFD arises from grain size distribution (σ_volume_), magnetocrystalline anisotropy dispersion (σ_Hk_) and misalignment of the c-axis(σ_axis_), and the extrinsic SFD comes from the grains’ dipolar and exchange coupling [4,17]. The standard deviation (σ_int_) of intrinsic SFD is 9.33 kOe (~21.2% of H_k_), estimated from the ΔH_int_/1.35 [4,17], and the (σ_int_)^2^ can be written as the summary of (σ_volume_)^2^ + (σ_axis_)^2^ + (σ_Hk_)^2^, which was the result of individual

Gaussian convolutions. The (σ_axis_) was estimated by fitting the rocking curve FePt (002) peak measured by XRD (Figure 1c–f), and the value is 4.55 kOe (~10.3% of H_k_) after Gaussian fitting [16].

The magnetization data were fitted to the modeled reversal curve in Equation (1) derived from reference [17], and the (σ_volume_) was extracted with the value of 1.23 kOe (~2.80% of H_k_).

M(H) = 1/2Erfc(x)

(1) $$x=\left\{\sigma_{v}^{2}-\ln\left[\left(\frac{1}{V}\right)\ln\left(\frac{f_{0}t}{\ln2}\right)\left(\frac{k_{B}T}{K_{u}}\right)\left[\frac{1}{{\left(1-\frac{{\mathrm{HM}}_{s}}{2{\mathrm{cK}}_{u}}\right)}^{2}}\right]\right]\right\}\left[\frac{1}{\sqrt{2\sigma_{v}}}\right]$$

Finally, the magnetic anisotropic dispersion (σ_Hk_)^2^ can be estimated by (σ_int_)^2^ − (σ_volume_)^2^ − (σ_axis_)^2^, and the value of (σ_Hk_) was 8.05 kOe, which is around 18.3% of the magnetic anisotropy field (H_k_ = 2K_u_/M_s_ = 44.0 kOe). As compared to the misalignment of the c-axis and the grain size distribution, the magnetic anisotropy dispersion contributes to a higher intrinsic SFD. Base on the magnetic interactions in the perpendicular media [4,17], the ΔH_ext_ can be used to derive the magnetic cluster size (D_n_), and the value of D_n_ was 46.6 nm in this sample. Equation (2) and (3), approximated from reference [18], were used to calculate the magnetic cluster size.

H_ext_ = H_in_ ~ H_ms_ − long(D_n_) (2)

H_ms-long_ = −[1 + (D_n_/t_mag_)^2^]^−^^1/2^M_mag_ (3)

In Equation (2), the extrinsic switching field distribution can be estimated by the internal effective field (H_in_) acting on the selected target grains. The H_in_ includes the shape anisotropy field from the columnar grains (the term was neglected in Equation (2)), the total exchange field (H_ex_) between the grains, and the magnetostatic field, which is divided into two terms, the local magnetostatic term (H_ms-local_) and the long-range magnetostatic term (H_ms-long_). The H_ex_ is balanced by H_ms-local_, and the sum of these two terms is zero. Finally, we consider the long-range magnetostatic field as the main contribution of the extrinsic switching field, and the magnetic cluster size can be estimated by Equation (3). The magnetic cluster is larger than the average grains size (28.2 nm) and grains pitch (3.97 nm) [16]. This indicates that there is a part of inter-granular coupling between grains. Table 1 lists and compares the magnetic switching field behavior of the FePt(B, Ag, C) granular film and reference sample [17]. The FePt(B, Ag, C)(8 nm) film shows a lower intrinsic field distribution than the reference FePt(Ag, C) film. It is suggested that the diffused B and N atoms improve the distribution in grain size, magnetocrystalline anisotropy and orientation. However, larger magnetic clusters and grains size were obtained after the high-temperature deposition of FePt(Ag, C, B) film.

The temperature dependency of coercivity was fitted by using the universal relation (seen in Equation (4)) to further understand the magnetization reversal process of the L1_0_ FePt(B, Ag, C) film in detail [19,20,21].

H_c_(T) = 2αK_u_(T)/M_s_ − N_eff_M_s_(T) (4)

**Figure 10:**
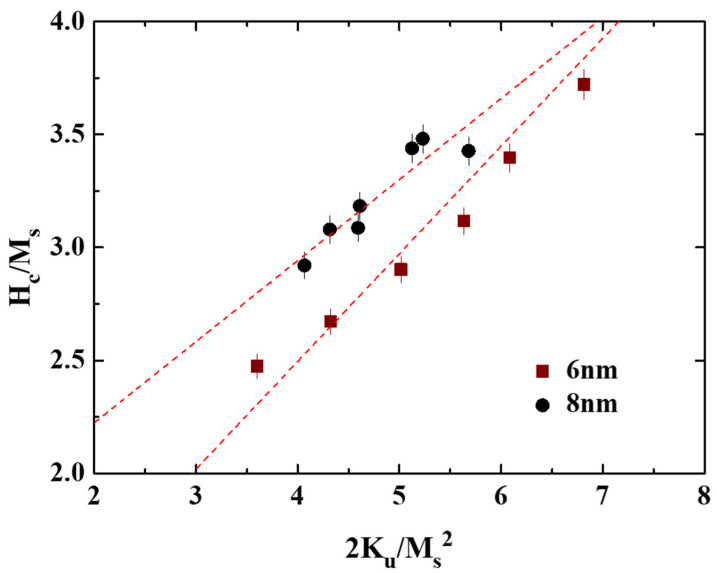
The microstructural parameter determination of the FePt(B, Ag, C)(8 nm)/MgTiON/CrRu film.

The experimental data plotted in Figure 2, Figure 3 and Figure 4 were used. The microstructural parameter was obtained as the slope of the linear fitting of H_c_/M_s_ versus K_u_/M_s_^2^ in Figure 10. The α values of the 8 nm and 6 nm samples are 0.36 and 0.58, respectively, and the maximum α value of 0.54, shown in reference [19], is 7 nm. The microstructure parameter is the convolution of reduced magnetocrystalline (α_K_), the misalignment grains (α_ψ_), and the grains exchange coupling (α_ex_). For parameter (α_ψ_), the rocking width shown in Figure 1c–f is smaller for L1_0_ FePt(B, Ag, C) films with a thickness of 6 nm, but the difference is only (0.2°/0.3°) for (001)/(002) diffraction peaks.

For parameter α_K_, the 6 nm thick FePt(B, Ag, C) film shows a higher K_u_ as compared to the 8 nm sample. For parameter α_ex_, the slightly soft Fe–B phase was observed (tiny kink in magnetization curves measured from 40 to 350 K) in the 8 nm sample, and the magnetic cluster (coupled grains) was observed in the TEM images (Figure 7d). It is suggested that an imperfect grains morphology always existed in the 6 nm and 8 nm samples. After combining all the factors, the lower microstructure parameter for the 8 nm sample can be explained.

## 4. Conclusions

The FePt(B, Ag, C) granular film prepared from the surface diffusion of an ultrathin B_4_C capping layer shows high perpendicular magnetic anisotropy and out-of-plane coercivity. After high-temperature deposition and post-annealing, the FePt grains are coarse, with an average size of 28.2 nm. Due to the part completion of intergranular coupling, the estimated magnetic cluster size was 46.6 nm, which is similar to the observed diameter from the TEM images. The lower microstructural parameter fitted from the temperature dependence of coercivity was also evidenced in the imperfect grains morphology. To correlate the microstructural grains, the intrinsic (ΔH_int_ = 12.6 kOe) and extrinsic switching field distributions (ΔH_ext_ = 1.62 kOe) were characterized by major and minor loops.

## Figures and Tables

**Table 1:** Magnetic switching field behavior of the FePt(B, Ag, C) granular film and the reference sample [17].

	**Δ** **Hint (kOe)**	**σ** **_axis_** **(kOe)**	**σ** **_vol_** **(kOe)**	**σ** **_Hk_** **(kOe)**
Reference	20.2	6.59	3.70	12.9
FePt/MgTiON	12.6	4.55	1.23	28.2
	**σ** **_int_ (kOe)**	**Δ** **Hext (kOe)**	**Cluster Size (nm)**	**Grain Size (nm)**
Reference	15.0	3.00	14.0	7.00
FePt/MgTiON	9.33	1.62	16.6	28.2

## Data Availability

Data available on request due to restrictions eg privacy or ethical. The data presented in this study are available on request from the corresponding author. The data are not publicly available.
